# Automatic failure mode evaluation using non-linear phase contrast correction to improve flow measurement accuracy in cardiovascular magnetic resonance phase contrast imaging

**DOI:** 10.1016/j.jocmr.2025.101895

**Published:** 2025-04-10

**Authors:** Ana Beatriz Solana, Savine C.S. Minderhoud, Piotr A. Wielopolski, Juan Antonio Hernandez-Tamames, Ricardo P.J. Budde, Willem A. Helbing, Martin A. Janich, Alexander Hirsch

**Affiliations:** a ASL Europe, GE HealthCare, Munich, Germany; b Department of Cardiology, Cardiovascular Institute, Thorax Center, Erasmus Medical Center, Rotterdam, the Netherlands; c Department of Radiology and Nuclear Medicine, Erasmus Medical Center, Rotterdam, the Netherlands; d Imaging Physics Department, Applied Physics Faculty, TU Delft, Delft, the Netherlands; e Department of Pediatrics, Division of Pediatric Cardiology, Erasmus Medical Center, Rotterdam, the Netherlands

**Keywords:** Background phase correction, Magnetic resonance imaging, Static phantom, Phase contrast velocity imaging

## Abstract

**Background:**

Phase contrast (PC) cardiovascular magnetic resonance (CMR) is clinically used to quantify flow. The quantification accuracy is diminished by background phase errors. Image-based background phase correction algorithms are commercially available, but their accuracy is still under evaluation. Here, we validate a recently developed non-linear phase contrast correction (nPCcor) algorithm that includes automatic failure mode classification in a large single-vendor multi-scanner retrospective study.

**Methods:**

Three hundred forty-six through-plane PC images at the aortic valve (AAo) and pulmonary artery (PA) were acquired on three different GE HealthCare 1.5T clinical MRI scanners. Each PC scan was repeated on a static phantom, and the static phantom-corrected PC series was considered as the reference standard. Two image-based static tissue background phase corrections were applied on each PC series: a linear and the nPCcor. Accuracy of nPCcor was studied by comparing the net flow in the vessel of interest for the uncorrected, linear-corrected, and nPCcor images with respect to the static phantom-corrected series. Accuracy was defined as a difference in net flow ≤10% with respect to the static phantom corrected net flow.

**Results:**

Flow measurements using the nPCcor images after nPCcor automatic classification were found to be accurate for 87% (281/323) of PC datasets, 6% and 17% better than using uncorrected and linear-corrected (p<0.05), respectively. Most importantly, nPCcor was able to correctly identify 70% (16/23) PC cases likely to provide inaccurate flow measurements. Flow measurements after nPCcor in the scanner with the largest phase offsets were found to be accurate for 74% (62/84) of PC datasets, 22% better than using the uncorrected images (p<0.05). nPCcor correction was statistically significant more accurate than linear correction for all scanners (p<0.05). The percentage of regurgitation reclassification of ≥1 category decreased to 8% (8/323) after nPCcor correction, 3% better than for uncorrected images.

**Conclusion:**

nPCcor with automatic failure mode evaluation improved accuracy with respect to no correction and linear correction and successfully identified PC scans that are likely to result in unreliable flow measurements. nPCcor performance and phase offset errors varied greatly among scanners using the same CMR protocol. nPCcor has higher impact in scanners exhibiting the largest background phase offsets.

**Trial registration:**

observational study

## Graphical abstract

**Figure:**
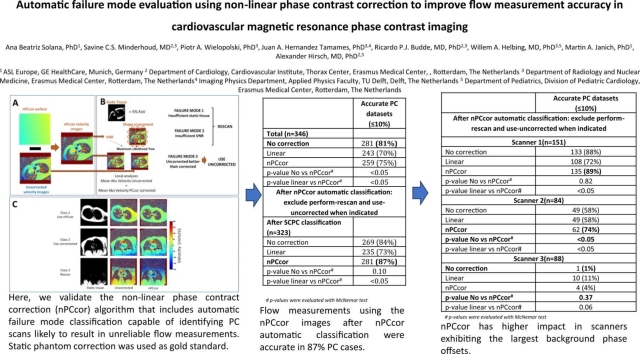

## 1. Introduction

Cardiovascular magnetic resonance (CMR) phase contrast (PC) imaging can be used to quantitatively and non-invasively measure velocity and flow in main arteries and veins of the heart [1], [2]. Clinically, 2D through-plane one-direction encoding PC imaging is the most commonly used CMR technique to evaluate regurgitation, stenosis, and shunts [3], [4].

However, PC CMR suffers from multiple sources of error that could diminish the clinical confidence in the reported results. In particular, background phase errors, which occur due to concomitant gradient fields, gradient non-uniformity or eddy-current effects, have been intensively investigated in the literature [5], [6], [7]. While concomitant gradient fields and gradient non-uniformity can be calculated analytically [8], [9] and corrected in reconstruction or postprocessing, zeroth and first spatial order eddy-current induced phase errors can be minimized by gradient pre-emphasis [6]. However, even after all these corrections, residual background phase errors [7] remain in PC CMR, varying in magnitude and distribution across the image field of view (FoV) for different scan parameters and across different scanners [5], [10]. Therefore, this source of error is particularly challenging to correct.

Several correction approaches have been proposed to correct for residual background phase errors for one-direction encoding 2D PC CMR. Repeating the same PC sequence on a static phantom and subtracting the velocity images is considered the reference standard [11], [12], [13], [14], [15], [16], [17]. However, this approach impedes the clinical workflow. Alternatively, image-based static tissue correction methods identify pixels in the FoV that are not moving across the cardiac cycle and are used as input to a polynomial fit which is then subtracted from the original PC velocity images [11], [14], [15], [18]. By using image-based static tissue correction methods, increased accuracy in flow has been demonstrated in several single and multi-center studies [11], [15], [18], [19], above all, when outlier detection is applied to better identify the static tissue [20]. However, other studies have reported no clinically significant change in accuracy [12], [14]. We recently even showed that static tissue background phase correction using polynomial fitting from first up to third order worsened accuracy compared to no correction on different magnetic resonance imaging (MRI) scanner models [13]. These negative results were consistent across different polynomial fittings and different software packages.

Recently a non-linear phase contrast correction (nPCcor) algorithm has been developed [14] and implemented in the latest GE HealthCare MR software releases. This is also an image-based static tissue correction method, however, this nPCcor algorithm also includes an automatic failure mode evaluation, which aims to evaluate whether static tissue information can be appropriately used to perform accurate correction.

The aim of this study is to validate this newly developed nPCcor algorithm using the same single-vendor multi-scanner database from the retrospective study used by Minderhoud et al. [13]. First, the automatic failure modes obtained from the nPCcor algorithm are assessed by evaluating whether the nPCcor algorithm can properly identify PC datasets that provide accurate flow measurements. Second, flow quantification including net flow and regurgitation fraction, are compared between using nPCcor, linear correction, no correction and static phantom correction as reference standard.

## 2. Methods

### 2.1 Study population and image acquisition

This retrospective study used the same PC scans as previously published by co-authors of this work and details about the included study population and image acquisitions have been described previously [13]. In short, images from 175 adults and pediatric patients as well as healthy volunteers who underwent CMR on one out of three different 1.5T MRI scanners (GE Healthcare, Waukesha, Wisconsin) were included (Table 1). Patients with an implanted mechanical aortic or pulmonary valve were excluded to avoid image artifacts. A total of 346 PC images were used for further analyses: 175 aortic valve (AAo) planes and 171 pulmonary artery (PA) planes. End-expiration breath-hold through-plane retrospective electrocardiographically triggered PC FastCINE at the AAo and approximately 1 cm above the pulmonary valve proximal to the pulmonary bifurcation were acquired. Standard velocity encoding value was set at 180 cm/s, however, increased in gradual steps up to 500 cm/s if necessary. Those PC sequences were repeated immediately after the CMR examination on a static gel phantom, still using the electrocardiographically from the patient. Scanner specifications and PC sequence parameters can be found in Table 1. Ethics committee approval was waived by the institutional review board (MEC-2019–0155) as this was a purely retrospective study. Authors agree to make data supporting the results presented in this paper available upon reasonable request.

**Table 1:** Scanner specifications and acquisition parameters.

	Scanner 1 (n=152)	Scanner 2 (n=95)	Scanner 3 (n=99)
Scanner model	Signa Artist 1.5T	Discovery MR450 1.5T	Signa Explorer 1.5T
Software version	DV26.0	DV25.0	DV25.0
Bore size (cm)	70	60	60
Maximum gradient amplitude (mT/m)	44	50	33
Maximum slew rate (T/s/m)	200	200	120
TR (ms)	Minimum (5.5–6.1)	Minimum (4.7–6.2)	Minimum (6.0–6.5)
TE (ms)	Minimum (3.2–3.8)	Minimum (1.8–3.4)	Minimum (3.6–3.9)
Temporal resolution (ms)	20–42	19–45	17–42
Views per segment	4–6	4–6	4–6
Number of reconstructed phases per cardiac cycle	30	30	30
Slice thickness (mm)	7	7	7
Field of View (cm)	31–38	31–38	31–38
Phase FoV (%)	75–100	75–100	75–100
Flip angle (°)	20	20	20
Flow compensation	Yes	Yes	Yes
Flow optimization	Yes	Yes	Yes
VENC (cm/s)	180–500	180–500	180–450
Breathing State	End-expiration Breath-hold	End-expiration Breath-hold	End-expiration Breath-hold
ECG Triggering	Retrospective	Retrospective	Retrospective

Values are presented as numbers or strings.

The table shows each scanner specification and main PC sequence parameter ranges for the three 1.5T MRI scanners. 324 PC datasets were accelerated by a factor of 1.5 to 2 and used ASSET reconstruction. *TR* repetition time, *TE* echo time, *FoV* field of view, *VENC* velocity encoding, *ECG* electrocardiogram, *ASSET* array coil spatial sensitivity enc

### 2.2 Non-linear PC correction algorithm

The nPCcor algorithm [14] is a non-linear image-based correction method for PC Cine CMR images. In a previous publication, this method has been called Self Calibrated Phase Correction (SCPC) [14], while the inline implementation in GE HealthCare MRI scanners is called “2D Phase Correction” and runs as a postprocessing step after a PC CINE acquisition is acquired. This algorithm was developed using over 500 volunteer and patient PC CINE datasets from 10 different 1.5T and 3T GE HealthCare MRI scanners. For the development of this algorithm, the retrospective database of PC images described in this manuscript was not used.

**Fig. 1:**
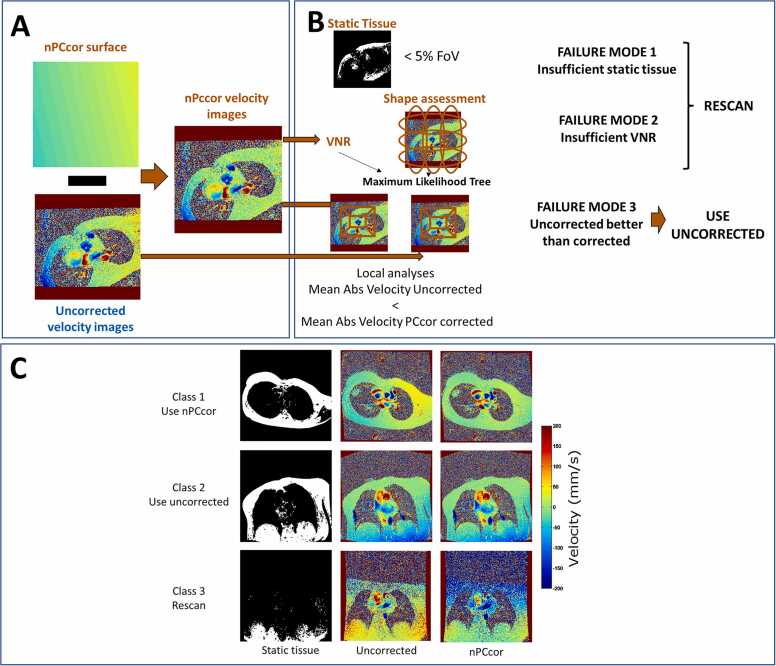
Schematic steps of nPCcor including automatic failure mode evaluation and dataset classification. A) nPCcor corrected velocity images are obtained by subtracting the non-linear nPCcor correction surface from each uncorrected velocity image along the cardiac cycle. B) Three failure modes are evaluated automatically. Failure mode 1 is flagged when insufficient static tissue is found. Failure mode 2 is identified when data have insufficient VNR. Failure mode 3 indicates that uncorrected images provide lower background phase errors in the proximities of the vessel of interest than nPCcor corrected images. C) Exemplary cases of PC scans automatically classified by nPCcor for: use-nPCcor-corrected class (when no failure modes were identified), use-uncorrected class (when only failure mode 3 was flagged) and perform-rescan class (if rescan is advised when failure mode 1 and/or 2 were detected). From left to right: the first column contains a binary mask indicating the automatically detected static tissue, the second and third column contain the mean velocity image over the cardiac cycle for the uncorrected and nPCcor corrected PC velocity images, respectively. *nPCcor* non-linear phase contrast correction, *VNR* velocity to noise ratio, *PC* phase contrast

Fig. 1 schematically shows the main steps of the algorithm. First, static tissue is identified automatically using the magnitude and the velocity images along all cardiac phases as input. Therefore, a global static tissue mask is created by identifying those pixels in the FoV with a mean magnitude value greater than 10% from the maximum and a standard deviation less than 7.5 cm/s along all cardiac phases. An initial linear fit [15] is then applied using this first global static mask. If the perpendicular vector between the linear fit and the scan plane is greater than 0.6 cm/s [5], the linear fit surface is subtracted from each velocity image, and the static tissue identification is restarted using these initial linear fit corrected velocity images. Note that this step is only performed to improve the identification of static tissue in the next step. An iterative outlier rejection (i.e., statistical iterative pruning, with four iterations [21]) is applied to the static tissue mask to remove pixels at tissue boundaries suffering from respiration-related, or flow-related ghosting artifacts. This will provide the final global static tissue mask for all cardiac phases. Additionally, a second static tissue mask (quiescent static tissue mask) is calculated only for the quiescent cardiac phase images. The quiescent static tissue mask contains only pixels that do not intercept with the global static mask and are removed from the mask. This increases the weighting of measurements from the quiescent cardiac phase images and identifies static voxels closer to the vessel of interest affected by flow artifacts in systole and, therefore, removed from the global static tissue mask. The mean velocity across the quiescent cardiac phases, instead of the mean velocity over all cardiac phases, is used for pixels within the quiescent static tissue mask. nPCcor fitting coefficients are found by polynomial regression (1) using the mean velocity across all cardiac phases inside the global static tissue mask and (2) using the mean velocity across the quiescent cardiac phases inside thequiescent static tissue mask. Five coefficients are estimated: zeroth-order, X, Y, Z linear terms (as nPCcor defines the 2D plane in the 3D space) and the Maxwell concomitant field shape (Eq. 1) as the fifth term.

(1) $${∆\emptyset }_{\mathrm{concomitant}}\left(x,y,z\right)=\frac{\gamma }{2B_{o}}\int_{0}^{t}\left[\frac{G_{z}{\left(t\right)}^{2}}{4}\left(x^{2}+y^{2}\right)+\left(G_{x}{\left(t\right)}^{2}\;+G_{y}{\left(t\right)}^{2}\right)z^{2}-G_{x}\left(t\right)G_{z}\left(t\right)\mathrm{xz}-G_{y}{\left(t\right)G}_{z}\left(t\right)\mathrm{yz}\right]\mathrm{dt}$$

where B_0_ corresponds to the main magnetic field of the scanner, γ is the gyromagnetic ratio, x, y, z are the positions in the 3D space, and G_x_, G_y_, G_z_ are the gradients applied by the pulse sequence which can be computed analytically and stored in the digital imaging and communications in medicine header of the PC images [8], [14].

The selection of the Maxwell concomitant field as fifth coefficient of the correction was based on the following three facts: (1) it has been shown using field probes that background phase error has non-linear components [7], (2) a full quadratic model requires the fitting of ten coefficients which could result in lack of robustness [20]; (3) last, experimental findings showed that the residual phase after linear correction resembled the shape of the concomitant field [14]. We hypothesized that Maxwell correction may be incomplete as it is based on the expected waveforms and not the actual gradients after eddy currents effects and compensation, which can lead to incomplete correction. Last, over-fitting is prevented by evaluating the created nPCcor fitting surface on a region of interest (ROI) of 4 cm of radius centered at the middle of the FoV (where the vessel of interest is expected to be located). If the average velocity in the ROI is lower than 0.6 cm/s, no correction is applied. Finally, as indicated in Fig. 1A the obtained non-linear nPCcor surface is then subtracted from each velocity image along all cardiac phases generating a new series of nPCcor PC images.

### 2.3 Failure modes and automatic classification

Once the fitting is completed, failure mode evaluation is started automatically (Fig. 1B). Three failure modes are defined as follows:

1) Failure mode 1 (insufficient static tissue)—This failure mode is flagged if the number of pixels of the addition of the global static tissue mask and the quiescent static tissue mask is <5% of all the pixels in the FoV.
2) Failure mode 2 (insufficient VNR)—Insufficient velocity-to-noise ratio (VNR) with curvature/slope failure prediction. A machine learning maximum likelihood decision tree predicting algorithm failure is created taking as inputs:
  a) VNR defined as the standard deviation of all pixels in the mean velocity across cardiac phases in the static tissue.
  b) the slope and curvature of the mean velocity across cardiac phases in the static tissue. These two parameters are calculated by dividing the mean velocity image into three horizontal and three vertical sections as indicated by the ellipses in Fig. 1B. Then the mean value inside each of those six sections (meanX (1,2,3) for horizontal, and meanY(1,2,3) for vertical) is computed. The first derivatives (dx1, dx2, dx for horizontal; dy1, dy2, dy for vertical) represent the slope, which is the rate of change of the mean values between sections. The second derivatives (ddx for horizontal; ddy for vertical) represent the curvature, which is the rate of change of the slope between sections.
3) Failure mode 3 (uncorrected better than corrected)—If the absolute mean velocity in static tissue (global and quiescent included) in an ROI in the center of FoV is equal or smaller before correction than after nPCcor, this failure mode is flagged. The smallest ROI centered in the FoV with 15% static voxels (representing the closest static tissue to the vessel of interest), is taken for the calculation. This failure mode can be seen as another layer to avoid correction over-fitting due to static tissue values far from the vessel of interest.

Based on those failure modes, each PC dataset is classified into the following three classes (Fig. 1C):

1) Use-nPCcor class. If no failure mode was identified, nPCcor images should be used for further analyses.
2) Use-uncorrected class. If only failure mode 3 was identified, uncorrected series should be used for further analyses.
3) Perform-rescan class. If failure mode 1 and/or failure mode 2 are identified, the algorithm suggests rescanning the PC dataset.

### 2.4 Analysis

PC flow measurements of the AAo and the PA were performed using Medis Qflow software (Medis Medical Imaging, Leiden, The Netherlands), using the same contours for the nPCcor, linear corrected, static phantom corrected, and uncorrected PC images. Linear correction was performed using the Medis algorithm. Net flow (ml/beat) was defined as the total forward flow minus total backward flow over all cardiac phases. Static phantom corrected data were used as reference standard and a net flow difference of >10% was considered clinically significant [13]. The percentage of PC datasets considered non-clinically significant with respect to static phantom correction was used as a measure of accuracy. Static phantom accuracy was evaluated as described in Hofman et al. [11] and only PC datasets with agreement within 0.6 cm/s between static tissue ROI in volunteer or patient PC data versus static phantom were included in the analyses [13]. We evaluated whether the nPCcor failure modes and automatic classification for each PC dataset (Fig. 1C) was successful (accurate) as follows:

- For use-nPCcor and use-uncorrected PC datasets: when net flow from nPCcor images differed ≤10% from phantom corrected net flow.
- For perform-rescan PC datasets: when net flow from nPCcor and uncorrected PC images differed >10% from phantom corrected net flow.

Additionally, we categorize the incorrectly automatically classified PC datasets into different types depending on the automatic classification results and the gold-standard classification using static phantom correction as reference. The details of this analysis can be found in the supplementary material.

The regurgitation fraction was calculated by dividing the backward flow by the forward flow as a percentage. AAo and PA regurgitation were graded as none (<5%), mild (5–20%), moderate (20–33% for the AAo, 20–40% for the PA, respectively), and severe (>33% for the AAo, >40% for the PA, respectively) [13], [22], [23]. The regurgitation fraction was graded using the static phantom correction data as the gold-standard. Reclassification after other correction methods was determined for each PC dataset.

### 2.5 Statistics

Mean ± standard deviation or median with interquartile range (IQR) were used to express continuous values for normal and skewed distributions, respectively. Categorical data are represented by percentages and frequencies. Paired t-tests or Wilcoxon signed rank tests were used to test for significant differences for continuous values that were normally or non-normally distributed, respectively. McNemar’s test was used to test for significant differences in the case of paired categorical data (e.g., differences between corrected and uncorrected data). Chi-square tests were used to test for significant differences in the case of unpaired categorical data (e.g, differences between vessels or scanners). P-values less than <0.05 were considered statistically significant. A weighted kappa was used to assess differences in regurgitation severity classification. Statistical analyses were conducted in Microsoft Excel (Microsoft Office 365 for Enterprise, Microsoft Inc, Redmond, Washington) using Real Statistics Resource Pack software (Release 7.6) and R Statistical Software (version 3.6.1, R Foundation for Statistical Computing, Vienna, Austria).

## 3. Results

**Fig. 2:**
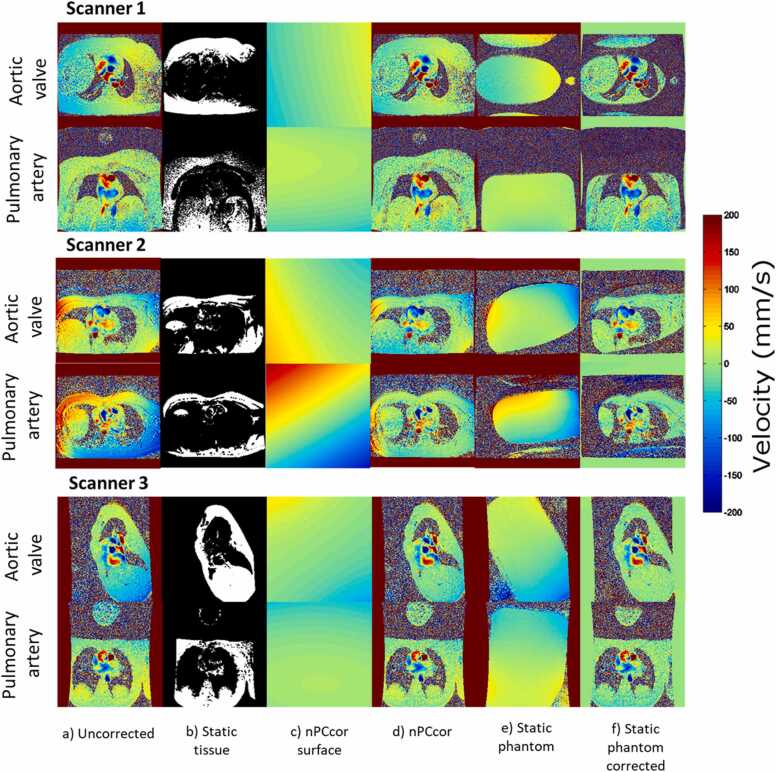
Aortic valve and pulmonary artery single-subject PC case for each scanner. Shown from left to right: a) the mean velocity across cardiac phases for the uncorrected PC series, b) the automatic static tissue for all cardiac phases (global static tissue mask plus static tissue during quiescent static tissue mask), c) the resultant nPCcor correction surface after applying the nPCcor algorithm, d) mean velocity across cardiac phases after nPCcor correction, e) mean velocity of the static phantom corresponding to the patient PC series, and f) mean velocity across cardiac phases after static phantom correction. *PC* phase contrast, *nPCcor* non-linear phase contrast correction

Exemplary images of the application of the nPCcor algorithm and static phantom correction for AAo and PA PC images of one representative patient at each MRI scanner are shown in Fig. 2. Fig. 2a illustrates the original, uncorrected, mean velocity of all cardiac phases. These images already show that scanner 2 has higher background phase errors compared to scanner 1 and scanner 3. This finding was already quantified in Minderhoud et al. [13], with uncorrected mean absolute velocity offsets (cm/s) of 0.7 (IQR 0.1–1.0), 1.4 (IQR 0.6–2.9) and 0.2 (IQR 0.1–0.4) for scanner 1, scanner 2 and scanner 3, respectively. Qualitatively, similarity between the nPCcor surface (Fig. 2c) and static tissue mean velocity is found indicating that nPCcor is indeed able to fit the background phase error. Furthermore, after nPCcor, the background phase error close to the center of the FoV (where the vessel of interest is found) is reduced. However, static phantom correction results in an additional reduction in the background phase error across the whole FoV even in areas far away from the center of the FoV.

### 3.1 Non-linear phase contrast correction automatic classification evaluation

Of the 346 PC datasets, the automatic classification of nPCcor identified 239 (69%) PC datasets as use-nPCcor corrected, 84 (24%) as use-uncorrected, and 23 (7%) as perform-rescan. The accuracy of the automatic classification of nPCcor is shown in Table 2. nPCcor provided correct automatic classification in 86% (297/346) of all PC images. Importantly, the nPCcor algorithm was able to correctly identify 70% (16/23 PC datasets that would likely produce unreliable flow measures (perform-rescan class). Of all PC images that were identified as use-uncorrected class by the nPCcor algorithm, 94% (79/84) were correctly identified. No statistically significant differences were found in the classification accuracy per vessel. However, there was a reduced classification accuracy for scanner 2.

**Table 2:** Evaluation of the accuracy of the nPCcor automatic classification.

	Accuracy of nPCcor automatic classification				
	All classes	Use-nPCcor class	Use-uncorrected class	Perform-rescan class	p-value*
Total	297/346 (86%)	202/239 (85%)	79/84 (94%)	16/23 (70%)	<0.05
Per vessel					
Aorta	151/175 (86%)	103/122 (84%)	43/46 (93%)	5/7 (71%)	0.16
Pulmonary artery	146/171 (85%)	99/117 (85%)	36/38 (95%)	11/16 (69%)	<0.05
p-value*	0.80	0.97	0.80	0.90	-
Per scanner					
Scanner 1	136/152 (89%)	81/96 (84%)	54/55 (98%)	1/1 (100%)	<0.05
Scanner 2	71/95 (75%)	55/73 (75%)	7/11 (64%)	9/11 (82%)	0.59
Scanner 3	90/99 (91%)	66/70 (94%)	18/18 (100%)	6/11 (55%)	<0.05
p-value*	<0.05	<0.05	<0.05	0.30	-

Values are presented as frequency (percentage)

* p-values were calculated with a Chi-square test

Statistical differences for each class, per vessel and per scanner were calculated using chi-square tests. Use-nPCcor are PC scans where no failure modes were found, use-uncorrected are PC scan where uncorrected was better than nPCcor and perform-rescan are PC scan where failure modes were found and rescan is advised.

*nPCcor* non-linear phase contrast correction

Of all PC datasets, 49/346 (14%) were incorrectly classified. However, 24/49 (49%) of those errors happened at scanner 2 where the nPCcor algorithm reduced the background phase error but did not meet the defined accuracy criteria (≤10% net flow difference with respect to static phantom correction). Another 10% (5/49) of the incorrectly classified PC datasets were from scanner 3 where signal-to-noise ratio and background phase error was lower and rescan was advised, however, net flow values were accurate with respect to static phantom correction. Further detailed results can be found in the supplementary material.

### 3.2 Net flow and regurgitation fraction

nPCcor slightly underestimated net flow compared to static phantom correction independent of scanner or location (AAo or PA) (Table 3, Table 4, supplementary material). nPCcor without including the failure mode evaluation, worsened the results with respect to no correction, similar to linear correction for all PC datasets together (Table 3), and when stratified by scanner for scanner 1 and scanner 3 (Table 4). nPCcor was statistically significantly better than linear correction for all PC datasets together (5% higher accuracy, Table 3) and for scanner 2 (14% higher accuracy, Table 4).

**Table 3:** Net flow changes without any background phase correction, after linear correction and after nPCcor with respect to static phantom correction for all PC datasets before and after nPCcor automatic classification.

	Net flow $ (mL/m^2^)	% Abs net flow difference versus phantom correction	Abs velocity difference versus phantom correction (cm/s)	Accurate PC datasets (≤10%)
Total (n=346)				
No correction	48 ± 13	3.3 (1.2–7.3)	0.5 (0.2–1.2)	281 (81%)
Linear Correction	47 ± 13	6.1 (3.0–11.2)	1.1 (0.6–1.7)	243 (70%)
nPCcor^	40 ± 13	5.2 (2.3–10.0)	0.9 (0.4–1.6)	259 (75%)
p-value No vs nPCcor^#^	<0.05	<0.05	<0.05	<0.05
p-value linear vs nPCcor^#^	0.21	<0.05	<0.05	<0.05
After nPCcor automatic classification: exclude perform-rescan and use-uncorrected when indicated *				
After nPCcor classification (n=323)*				
No correction	49 ± 13	3.2 (1.2–6.7)	0.5 (0.2–1.1)	269 (84%)
Linear Correction	47 ± 12	6.1 (3.0–10.5)	1.0 (0.6–1.6)	235 (73%)
nPCcor^	48 ± 12	3.9 (1.7–7.1)	0.6 (0.3–1.1)	281 (87%)
p-value No vs nPCcor^#^	<0.05	0.10	0.43	0.10
p-value linear vs nPCcor^#^	0.09	<0.05	<0.05	<0.05

Values are presented as mean ± standard deviation, median (IQR) or frequency (percentage)

*Perform-rescan PC datasets were excluded from analyses

^ Uncorrected net flow was used when nPCcor automatic classification indicated use-uncorrected PC datasets

# p-values were evaluated with paired-t tests, Wilcoxon signed rank and McNemar tests for frequency data.

$ Net flow divided by the Body Surface Area (BSA)

*nPCcor* non-linear Phase Contrast correction, *Abs* absolute

**Table 4:** Net flow changes without any background phase correction, after linear correction and after nPCcor with respect to static phantom correction for all PC datasets before and after nPCcor automatic classification stratified per scanner.

	Net flow^$^ (ml/m^2^)	% abs difference versus phantom correction	Abs velocity difference versus phantom correction (cm/s)	Accurate PC datasets (≤10%)	Net flow^$^ (ml/m^2^)	% Abs difference versus phantom correction	Abs velocity difference versus phantom correction (cm/s)	Accurate PC datasets (≤10%)	Net flow^$^(ml/m^2^)	% Abs difference versus phantom correction	Abs velocity difference versus phantom correction (cm/s)	Accurate PC datasets (≤10%)
	Scanner 1 (Total N=152)				Scanner 2 (Total N=95)				Scanner 3 (Total N=99)			
No correction	47±10	3.8 (1.9–6.3)	0.7 (0.3–1.0)	134 (88%)	51±17	8.9 (4.4–15.0)	1.4 (0.7–3.3)	49 (52%)	48±12	1.0 (0.5–2.3)	0.2 (0.1–0.4)	98 (99%)
Linear correction	47±11	6.5 (4.3–10.6)	1.2 (0.8–1.6)	109 (72%)	47±16	9.7 (4.1–18.2)	1.4 (0.9–2.3)	50 (53%)	47±12	3.3 (2.1–6.2)	0.6 (0.3–1.1)	84 (84%)
nPCcor	47±10	6.6 (3.4–10.5)	1.1 (0.6–1.6)	108 (71%)	48±15	6.3 (3.2–12.3)	1.1 (0.6–1.7)	64 (67%)	47±13	2.3 (1.0–6.1)	0.4 (0.2–0.9)	87 (87%)
p-value no vs nPCcor#	0.69	<0.05	<0.05	<0.05	<0.05	<0.05	<0.05	<0.05	<0.05	<0.05	<0.05	<0.05
p-value linear vs nPCcor#	0.54	<0.05	<0.05	1.00	0.07	<0.05	<0.05	<0.05	0.42	<0.05	0.33	0.58

After nPCcor automatic classification: exclude perform-rescan and use-uncorrected when indicated												

	Scanner 1 (N=151)*				Scanner 2 (N=84)*				Scanner 3 (N=88)*			
No correction	47±10	3.8 (1.9–6.3)	0.7 (0.3–1.0)	133 (88%)	51±16	7.8 (3.0–16.7)	1.4 (0.6–2.6)	49 (58%)	49±12	1.1 (0.6–2.2)	0.2 (0.1–0.4)	87 (99%)
Linear correction	46±11	6.8 (24.3–11.2)	1.2 (0.8–1.6)	108 (72%)	47±14	7.7 (4.0–13.8)	1.3 (0.8–2.1)	49 (58%)	47±12	3.3 (2.0–6.0)	0.6 (0.3–0.9)	78 (89%)
nPCcor^^^	47±10	4.1 (2.3–7.2)	0.7 (0.4–1.1)	135 (89%)	48±15	6.1 (3.2–10.3)	1.0 (0.6–1.5)	62 (74%)	48±12	1.8 (0.8–4.1)	0.3 (0.1–0.6)	84 (96%)
p-value No v nPCcor^#^	0.13	0.23	0.16	0.82	<0.05	<0.05	<0.05	<0.05	<0.05	<0.05	<0.05	0.37
p-value linear vs nPCcor^#^	<0.05	<0.05	<0.05	<0.05	0.13	<0.05	<0.05	<0.05	<0.05	<0.05	<0.05	0.06

Values are presented as mean ± standard deviation, median (IQR) or frequency (percentage)

* Perform-rescan PC datasets were excluded from analyses

*^ Uncorrected net flow was used when nPCcor automatic classification indicated use-uncorrected PC datasets*

*# p-values were evaluated with paired-t tests, Wilcoxon signed rank and McNemar tests for frequency data*

*$ Net flow divided by the Body Surface Area (BSA)*

*nPCcor* non-linear phase contrast correction, *Abs* absolute

After nPCcor automatic classification, 87% (281/323) of the PC datasets were accurate (net flow differed ≤10% with respect to phantom correction). This represents 6% more than without correction and failure mode evaluation (Table 3). There were not clinically significant differences in accuracy between nPCcor and no correction. However, there was a significant difference between nPCcor and linear correction (14% higher accuracy, Table 3). nPCcor had the largest impact on scanner 2, the scanner with the largest background phase errors (Table 4). With this scanner, net flow differed ≤10% with respect to phantom correction in 74% (62/84) of the PC series after nPCcor and automatic classification versus 52% (49/95) without correction (a difference of 22%). No statistically significant change in accuracy between nPCcor and no correction was obtained for scanner 1 or scanner 3. However, for scanner 3, there were statistically significant higher mean velocity difference errors for nPCcor than uncorrected, although small with median velocity error was 0.3 cm/s and 0.2 cm/s, respectively. After nPCcor automatic classification, nPCcor was more accurate than linear correction for scanner 1 (17% higher accuracy, Table 4) and scanner 2 (16% higher accuracy, Table 4) and decrease absolute net flow and velocity difference with respect to static phantom correction for the three scanners (Table 4).

Per vessel, an increased accuracy of 5% for AAo and 6% for PA PC cases after nPCcor with automatic classification was found with respect to no correction but this difference was not statistically significant. Per vessel, nPCcor performance was statistically significantly better than linear correction with and without nPCcor automatic classification (supplementary material).

Twenty-two out of the 246 PC datasets used in this work did not use ASSET reconstruction technique and presented mild FoV aliasing. However, the failure mode classification flagged 55% (12/22) of those PC datasets automatically in perform-rescan class, from the remaining ten PC datasets, only two PC datasets were found inaccurate (>10% difference in net flow with respect to static phantom correction) and not detected by the automatic failure mode detection.

Regurgitation was classified as none, mild, moderate, and severe, and the misclassification compared to the phantom corrected category is depicted in Table 5. There was only a statistically significant difference in 1 category change between nPCcor and linear correction regurgitation misclassification after nPCcor automatic classification. The percentage reclassification of ≥1 category compared to phantom corrected PC datasets was 10% (36/346) with no correction, 16% (54/346) with linear correction, and 14% (47/346) with nPCcor. However, after nPCcor automatic classification, the percentage reclassification of ≥1 category decreased to 8% (25/323) with nPCcor. This indicates that the nPCcor failure mode can identify PC datasets that are unreliable for regurgitation measurements.

**Table 5:** Regurgitation reclassification comparing no correction, linear correction, and nPCcor with respect to phantom correction for all phase contrast datasets before and after nPCcor automatic classification.

	1 category^a^	≥2 category^b^	Weighted Kappa (95% CI)
Total(N=346)			
No correction	33 (10%)	3 (1%)	0.86 (0.81–0.90)
Linear correction	46 (13%)	8 (2%)	0.78 (0.72–0.84)
nPCcor	40 (12%)	7 (2%)	0.81 (0.75–0.86)
p-value no vs nPCcor^#^	0.37	0.29	
p-value linear vs nPCcor^#^	0.34	1.0	
	1 category^a^	≥2 category^b^	Weighted Kappa (95% CI)
After nPCcor classification (n=323)*			
No correction	29 (9%)	0	0.89 (0.85–0.93)
Linear correction	39 (12%)	4(1%)	0.82 (0.76–0.87)
nPCcor^^^	24 (7%)	1	0.90 (0.86–0.94)
p-value no vs nPCcor^#^	0.44	-	
p-value linear vs nPCcor^#^	<0.05	0.25	

Values are presented as frequency(percentage) or confidence interval

* Perform-rescan PC datasets were excluded from analyses

*^ Uncorrected net flow was used when nPCcor automatic classification indicated use-uncorrected PC datasets*

*# p-values were evaluated with McNemar test for frequency data.*

*^a^ indicates number of studies in which regurgitation severity shifted only one category (e.g. from mild to moderate);*

*^b^ number of studies in which regurgitation severity shifted with two categories or more (e.g. from none to moderate or mild to severe).*

*CI* confidence interval, *nPCcor* non-linear phase contrast correction

## 4. Discussion

This study validated a novel, fully automated background phase correction method for PC CINE MRI called nPCcor. We observed that nPCcor improves flow measurement accuracy, with the greatest benefit in scanners with larger background phase errors. Importantly, nPCcor incorporates an automatic failure mode evaluation that identifies unreliable PC scans.

Background phase errors in 2D PC CMR have been investigated over the last 30 years [7], [8], [14], [15], [16]. Repeating the clinical PC scan on a static phantom [16] or using dynamic field probes [7] to calculate the background phase errors, appear to reduce the error below a clinically significant threshold [5], [13]. However, these techniques either impede the workflow or require additional hardware.

The use of image-based static tissue algorithms is very attractive in clinical practice, as the postprocessing is performed on reconstructed velocity and magnitude PC images. However, studies have shown contradictory results. A recent multicenter multi-vendor study [11] claimed that image-based background phase correction had comparable efficacy to static phantom-based background phase correction. On the other hand, Rigsby et al. [12] and Paul et al. [14] have reported minimal clinical impact on Qp:Qs with and without image-based background phase correction methods in congenital cardiac disease populations. Both studies observed residual error in Qp:Qs quantification after background phase correction and no significant difference with no corrected based Qp:Qs. Ultimately, we have previously shown that image-based background phase correction worsened the net flow, Qp:Qs and regurgitation classification measures compared to phantom correction [13]. These contradictory results could also be explained due to incorrect identification of static tissue, like the presence of FoV aliasing (wrapping) artifact [20], [24], however, FoV aliasing artifacts only provided inaccurate results in 2 out of 346 PC datasets in our database.

The nPCcor algorithm belongs to the group of “fully-automated image-based static tissue background phase correction” algorithms [12], [20], [24]. Like nPCcor, the works from Pruitt et al. [24] and Fischer et al. [20] included an outlier rejection step when identifying static tissue. However, those two algorithms could also eliminate wrapping around static tissue. On the other hand, while Pruitt et al. [24] used a second-order fitting, Fischer et al. [20] showed better performance using first-order correction. In our study, we introduce two main novelties with nPCcor as follows: 1) a second-order spatial fit term based upon the field shape of the Maxwell concomitant field, and 2) an automatic failure mode detection.

However, the addition of the second-order spatial fit term alone is only statistically significantly different from linear correction implemented in Medis cardiac postprocessing software for one of the scanners in the study (scanner 2). However, we have clearly shown the benefit of adding a failure mode evaluation that identifies PC scans in which image-based background correction will likely fail. None of the studies discussed above contained such a failure mode evaluation step. Furthermore, we observed a large variability in the performance of the background phase error correction algorithm per scanner. Scanners with small background phase errors do not need any background correction and applying such a correction can worsen accuracy, while scanners with large background phase errors will benefit from background phase correction algorithms. We have proposed an automatic failure mode evaluation that assesses whether the amount of identified static tissue and VNR in the PC images is sufficient. Here, we have combined this failure mode evaluation with the nPCcor algorithm [14], but it could be adapted to any image-based background phase correction algorithm pipeline.

The accuracy of that PC datasets automatic classification was based on the net flow quantification of the vessel of interest comparing, the uncorrected, the nPCcor, the linear correction and the static phantom corrected PC images. Static phantom correction was used as reference standard [16]. It is known that static phantom background phase offset can differ from clinical PC scans due to coil load differences, temperature changes or scanner drifts [10]. To assure static phantom PC dataset quality, the same method proposed by Hofman et al. [11] was applied from the original database [13]. The velocity in an ROI in the static tissue of the PC clinical dataset was compared to the same ROI in the static phantom PC image. The clinical PC dataset was discarded for further analyses if a mismatch of more than 0.6 cm/s was found. All PC datasets included in this study passed that test.

Our automatic classification was correct in 86% (297/346) of the PC cases and there were no statistically significant differences per vessel. This means that both the nPCcor algorithm and the failure mode evaluation perform similarly for different plane prescription obliquities. However, there were statistically significant differences per classification category and per scanner. Use-nPCcor class accurately identified 85% (202/239) of the PC cases. Errors in the classification were due to two reasons as follows: 1) The background phase error was too large that the algorithm was able to reduce but not enough to reach our clinically significant net flow difference criterion. 2) Our automatic local evaluation failed to detect that uncorrected images were better than nPCcor images. In these cases, the main reason was that static tissue found in the PC image was far from the vessel of interest. Use-uncorrected class was identified properly in 94% (79/84) of the PC cases, meaning that the local evaluation of the accuracy of the fitting is efficient when the uncorrected images are better than the nPCcor images. Last, perform-rescan classified only 70% (16/23) of the PC cases correctly. Perform-rescan PC datasets were either noisy or had a FoV that was too small. In these cases, the algorithm did not find enough static tissue to perform and/or evaluate the accuracy of the correction. This does not mean that a PC dataset depicted as perform-rescan is automatically incorrect. However, without enough static tissue the algorithm simply cannot evaluate it. This explains why 7 out of the 23 PC cases from perform-rescan class were providing accurate net flow measures at the vessel of interest.

Three different 1.5T MRI scanners were used in this study. Statistically significant differences among scanners were found in the accuracy of the classification for use-nPCcor and use-uncorrected classes. This can be explained by the differences in net flow accuracy with respect to static phantom correction for each scanner. The results indicate that using nPCcor is beneficial in scanner 2, but does not have a clinical impact in scanner 1 and scanner 3. This also suggests that the development of a calibration approach that is scanner specific and perhaps even protocol specific, would be beneficial in clinical practice. Such a calibration could activate or deactivate background phase correction for specific configurations. Additionally, the use of deep learning denoising reconstruction [25] may improve the performance of the algorithm as it will boost the signal-to-noise ratio. We hypothesized that this would have a higher impact in lower gradient performance systems, such as scanner 3.

It has been suggested that different polynomial orders should be used for different scanners [11], [13] to improve accuracy. Minderhoud et al. [13] showed that image-based background phase correction even with optimized polynomial order per scanner worsened the net flow results with respect to no correction taking static phantom corrected as reference. However, on the same PC scans, nPCcor improved accuracy by 6% with respect to no correction. This improvement is partially due to the automatic failure evaluation feature of the nPCcor algorithm. Additionally, higher-order polynomial fitting did not show accuracy improvement in previous works [11], [18]. We hypothesize that it may not be robust to all PC scans of a given scanner due to insufficient or noisy static tissue.

Finally, nPCcor does not impact the clinical practice workflow because it is fully automatic and runs on the MRI scanner as an inline postprocessing task called “2D PC Correction” and provides feedback on the accuracy of the algorithm in real-time. This allows the scanner operator to rescan a specific PC scan if the automatic algorithm identifies the PC scan as likely to produce inaccurate velocity and net flow values.

## 5. Limitations

The nPCcor fully automatic algorithm may fail in the presence of FoV aliasing (wrap-in artifact), where the static tissue identified does not belong to the spatial location and hence its velocity should not be used. Instead, FoV aliasing is prevented in the PC scans by the vendor provided double-FoV coil combination method ASSET (Array coil Spatial Sensitivity Encoding). Moreover, the nPCcor algorithm could also be extended to include FoV aliasing removal techniques [20], [24]. Also, nPCcor algorithm assumes that the vessel of interest is in the center of the FOV. While reasonable for single-slice PC scans, it could be improved by incorporating deep learning-based automatic vessel segmentation. Last, one of the main novelties of nPCcor is using the Maxwell concomitant field in the fitting based on experimental findings showing that the residual phase after linear correction resembled the shape of the concomitant field [14]. The origin of this experimental finding across different MRI scanners should be studied using field probes cameras. This study was performed on a dataset of existing PC images and lacks the clinical impact of the automatic suggestion to rescan when PC scans are likely to produce inaccurate flow results are detected. A prospective study is needed to evaluate whether the rescanning suggestions can produce correct PC datasets. Here, we only analyzed AAo and PA PC scans, but no significant performance differences were observed. We expect these results to be replicated in other vessels.

The study data comes from a single site using only 1.5T GE Healthcare scanners, with heterogeneous results across scanners. Each scanner was a different type with varying gradient coils, which can influence background phase errors. Apart from replicating these findings in a multi-center multi-vendor study, it will also be of interest to evaluate whether scanners of the same type have the same performance In fact, in a multicenter multi-vendor study conducted by Hofman et al. [11], there were two scanners of the same type which showed significant differences in the velocity offset. This may be due to the variations in pre-emphasis calibration of each scanner [6], which can affect the residual background phase errors.

This work used clinical PC scans using the same CMR protocol parameters at each scanner and using only breath-hold acquisitions. Another study including both breath-hold and free-breathing scans should be conducted to test the nPCcor algorithm in those conditions, including the presence of breathing artifacts. Moreover, as differences in PC sequence parameters like echo time or repetition time [26] or differences in the flow encoding waveforms [11], [27] affect the shape and strength of background phase errors, testing different protocols at each scanner would be very valuable It would also be advisable to conduct a follow-up study to assess the impact of different imaging protocols on the performance of the nPCcor algorithm.

## 6. Conclusions

The accuracy of automatic image-based static tissue background phase correction algorithms depends on the quality of the static tissue identified and varies greatly between scanners. The nPCcor significantly improved net flow results (by 22% with respect to no correction) in the scanner with the largest background phase offsets, while it had minimal impact on the other scanners in the study. Importantly, the nPCcor algorithm includes an automatic failure mode evaluation that can identify PC scans likely to result in unreliable flow measurements. This ability to prevent unreliable data is crucial as clinical decisions are often based on these individual measurements.

## Funding

Thorax Foundation: The Thorax Foundation had no influence on study design; in the collection, analysis, and interpretation of data; in the writing of the report; or the decision to submit the article for publication.

## Author contributions

A.B.S. and A.H. designed the study. S.C.S.M., W.A.H. and A.H. performed the clinical CMR scanning. A.B.S., S.C.S.M. and A.H. performed the flow measurements analysis. A.B.S., S.C.S.M. and A.H. performed the statistical data analyses. A.B.S. and A.H. drafted the manuscript. All authors contributed to the manuscript and read and approved the final version.

## Ethics approval and consent

Since this is a purely observational and retrospective study, the need for ethics committee approval was waived by the institutional review board (MEC-2019–0155). All healthy volunteers provided informed consent.

## Consent for publication

Not applicable.

## Declaration of competing interests

The authors declare the following financial interests/personal relationships which may be considered as potential competing interests: Alexander Hirsch, Ana Beatriz Solana, Martin A. Janich, and Juan Antonio Hernandez-Tamames report that financial support was provided by GE HealthCare. Alexander Hirsch reports a relationship with GE HealthCare that includes funding grants. Ana Beatriz Solana and Martin A. Janich report a relationship with GE HealthCare that includes employment. Juan Antonio Hernandez-Tamames reports a relationship with GE HealthCare that includes funding grants. Alexander Hirsch is a member of the medical advisory board of Medis Medical Imaging Systems. Other authors declare that they have no known competing financial interests or personal relationships that could have appeared to influence the work reported in this paper.

## Data Availability

The datasets used and/or analyzed supporting the conclusions of the article are available from the corresponding author upon reasonable request.
