# Supplementary material for: Automatic failure mode evaluation using non-linear phase contrast correction to improve flow measurement accuracy in cardiovascular magnetic resonance phase contrast imaging
Source: J Cardiovasc Magn Reson. 2025 Apr 10;27(1):101895. doi: 10.1016/j.jocmr.2025.101895 (PMC12182816; doi:10.1016/j.jocmr.2025.101895)
Supplement: Supplementary file 1 — Supplementary material [file mmc1.docx]

**Supplementary data**

**Automatic failure mode evaluation using non-linear phase contrast correction to improve flow measurement accuracy in cardiovascular magnetic resonance phase contrast imaging**

Ana Beatriz Solana, PhD^1^, Savine C.S. Minderhoud, MD^2,3^, Piotr A. Wielopolski, PhD^3^, Juan A. Hernandez Tamames, PhD^3,4^, Ricardo P.J. Budde, MD, PhD^2,3^, Willem A. Helbing, MD, PhD^3,5^, Martin A. Janich, PhD^1^, Alexander Hirsch, MD, PhD^2,3^

**Affiliations:**

^1^ ASL Europe, GE HealthCare, Munich, Germany

^2^ Department of Cardiology, Cardiovascular Institute, Thorax Center, Erasmus Medical Center, , Rotterdam, The Netherlands

^3^ Department of Radiology and Nuclear Medicine, Erasmus Medical Center, Rotterdam, The Netherlands

^4^ Imaging Physics Department, Applied Physics Faculty, TU Delft, Delft, The Netherlands

^5^ Department of Pediatrics, Division of Pediatric Cardiology, Erasmus Medical Center, Rotterdam, The Netherlands

**Methods – Non-linear phase contrast correction (nPCcor) automatic classification error types**

We categorize the incorrectly automatically classified phase contrast (PC) datasets into four different types:

- Type 1 (should-use-uncorrected): use-nPCcor class was automatically identified but it should have been use-uncorrected-class. This means that net flow using the uncorrected PC series yielded ≤10% differences with respect to static phantom corrected net flow, but nPCcor images net flow differed >10% from static phantom corrected net flow.
- Type 2 (should-rescan): use-nPCcor or use-uncorrected classes were automatically identified but they should have been should-rescan class. This type indicates that both uncorrected and nPCcor images net flow differs >10% from static phantom corrected net flow. Rescan should have been advised.
- Type 3 (should-use-nPCcor): use-uncorrected class was automatically identified but it should have been use-nPCcor class. In this case, the nPCcor corrected net flow differed ≤10% with respect to the static phantom net flow but the uncorrected net flow was >10%.

Type 4 (should-no-rescan): perform-rescan class was automatically identified but it should have been use-nPCcor or use-uncorrected classes. Rescan was advised but uncorrected and/or nPCcor images net flow was ≤10% different from static phantom corrected net flow.

**Supplementary Figure 1:**

This figure describes the types of error of the automatic classification of the nPCcor algorithm: Type 1 (should-use-uncorrected): use-nPCcor class was automatically identified but it should have been use-uncorrected class; Type 2 (should-rescan): use-nPCcor or use-uncorrected classes were automatically identified but it should have been perform-rescan class; Type 3 (should-use-nPCcor): use-uncorrected class was automatically identified but it should have been use-nPCcor class; Type 4 (should-no-rescan): perform-rescan class was automatically identified but it should have been use-nPCcor-corrected or use-uncorrected classes.


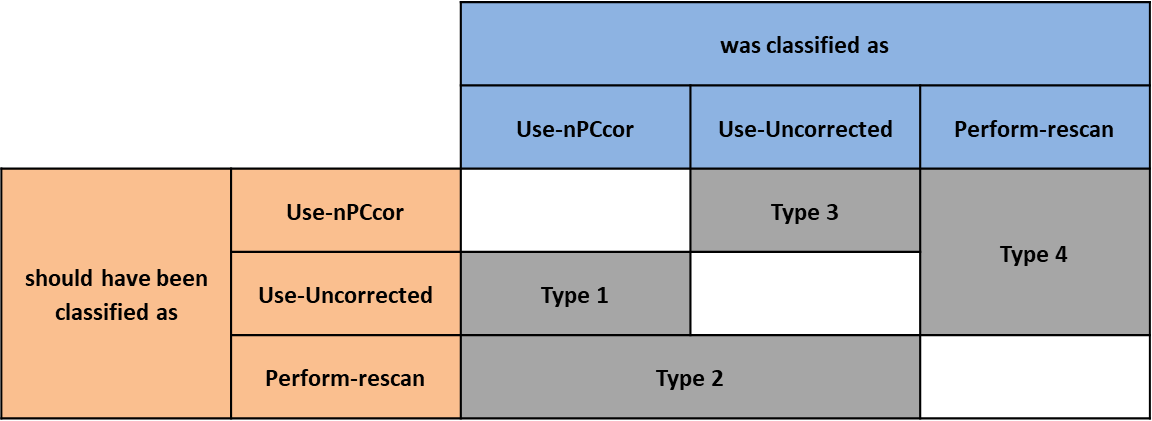


**Results - nPCcor automatic classification error types**

Of all PC datasets, 49/346 (14%) were incorrectly classified of which 17 (35%) were type 1 (should-use-uncorrected) errors, 24 (49%) were type 2 (should-rescan), 1 (2%) was type 3 (should-use-nPCcor), and 7 (14%) were type 4 (should-no-rescan). Supplementary file 2 shows the types of errors stratified by vessel and by scanner. Most likely, type 1 errors are the most critical where nPCcor worsened the net flow results with respect to no correction. 2 out of 17 were from scans with FoV aliasing. We performed a sensitivity analysis using a threshold of 15% difference in net flow of the nPCcor versus static phantom correction (instead of 10%), only 5 out of 17 (30%) PC series had a difference of >15%. Rescanning was advised in 23 PC images, however, this was unnecessary (type 4 error) in 7 scans (30%). Five out of these 7 incorrectly classified scans were from scanner 3 where image quality and background phase error were lower with respect to the other two scanners.

**Supplementary Figure 2**: **Evaluation of the type of failed automatic classification.**

The number of failed automatic nPCcor classification is shown for the total number of phase contrast (PC) scans (A), and further stratified per vessel (B) and per scanner (C). Type 1 (should-use-uncorrected) errors mean that nPCcor was selected for use in further analyses, but no correction should have been selected. Type 2 (should-rescan) errors represent the PC dataset that should have been classified to be rescanned, but they were not. Type 3 (should-use-nPCcor) errors indicate that nPCcor was better than no correction, but no correction was suggested to be used for further analyses. Last, type 4 (should-no-rescan) error occurred when the PC acquisition was indicated to be rescanned, but it was not necessary. Pie plots contain the number of errors that they represent in value (n) and in percentage. Accordingly, pie plots are also proportionally scaled in diameter.


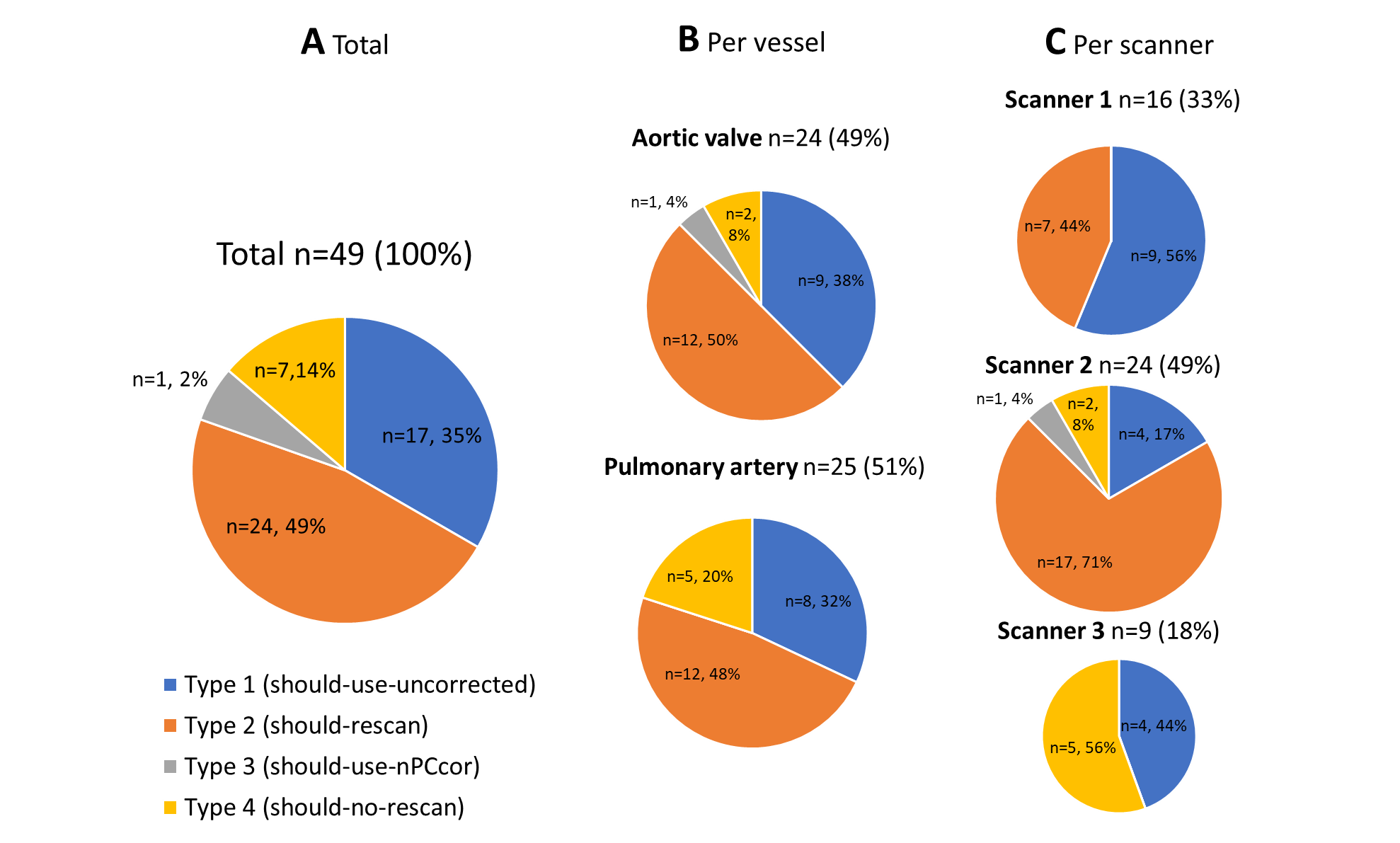


**Supplementary Table 1**: Net flow changes without any background phase correction, after linear correction, and after non-linear phase contrast correction (nPCcor) with respect to phantom correction for all phase contrast datasets before and after nPCcor automatic classification stratified per vessel.

|  | **Net flow $  (ml/m^2^)** | **% Abs difference versus phantom correction** | **Abs velocity difference versus phantom correction (cm/s)** | **Accurate PC datasets  (≤10%)** | **Net flow $  (ml/m^2^)** | **% Abs difference versus phantom correction** | **Abs velocity difference versus phantom correction (cm/s)** | **Accurate PC datasets  (≤10%)** |
| --- | --- | --- | --- | --- | --- | --- | --- | --- |
| **Total (n=346)** | **Aortic valve (Total N=175)** | | | | **Pulmonary valve (Total N=171)** | | | |
| No correction | 50 ± 14 | 3.4 (1.1-7.6) | 0.6 (0.2-1.2) | 141 (80%) | 47 ± 12 | 3.2 (1.3-6.9) | 0.5 (0.2-1.1) | 140 (82%) |
| Linear correction | 49 ± 13 | 5.0 (2.8-11.2) | 1.1 (0.7-1.6) | 126 (72%) | 45 ± 11 | 5.7 (2.6-10.0) | 0.9 (0.5-1.7) | 117 (68%) |
| nPCcor | 49 ± 14 | 5.3 (2.1-10.5) | 0.9 (0.5-1.5) | 124 (73%) | 45 ± 11 | 5.2 (2.4-9.7) | 0.9 (0.3-1.6) | 131 (77%) |
| p-value no vs nPCcor^#^ | 0.22 | <0.05 | <0.05 | 0.08 | <0.05 | <0.05 | <0.05 | 0.20 |
| p-value Linear vs nPCcor^#^ | 0.10 | <0.05 | <0.05 | <0.05 | <0.05 | <0.05 | <0.05 | <0.05 |
| **After nPCcor automatic classification: exclude perform-rescan and use-uncorrected when indicated*** | | | | | | | | |
| **Total (n=323)*** | **Aortic valve (Total N=168)*** | | | | **Pulmonary valve (Total N=155)*** | | | |
| No correction | 48 ± 11 | 3.4 (1.3-7.1) | 0.6 (0.2-1.1) | 142 (85%) | 48 ± 11 | 3.1 (1.2-6.6) | 0.5 (0.2-1.1) | 127 (82%) |
| Linear correction | 48 ± 13 | 5.0 (2.5-10.5) | 1.1 (0.7-1.6) | 121 (73%) | 45 ± 13 | 5.3 (2.5-10.2) | 0.9 (0.4-1.6) | 114 (74%) |
| nPCcor^ | 47 ± 11 | 3.7 (2.1-7.1) | 0.7 (0.3-1.2) | 146 (87%) | 48 ± 11 | 4.0 (1.6-7.1) | 0.6 (0.3-1.1) | 135 (87%) |
| p-value no vs nPCcor^#^ | <0.05 | 0.11 | 0.09 | 0.52 | <0.05 | 0.12 | 0.13 | 0.15 |
| p-value Linear vs nPCcor^#^ | <0.05 | <0.05 | <0.05 | <0.05 | <0.05 | <0.05 | <0.05 | <0.05 |

*Values are represented as mean ± standard deviation, median (IQR), or frequency (percentage), respectively.*

** Perform-rescan PC datasets were excluded from analyses*

*^ Uncorrected net flow was used when nPCcor automatic classification indicated use-uncorrected PC datasets*

*# p-values were evaluated with paired-t tests, Wilcoxon signed rank, or McNemar tests for frequency data.*

*$ Net flow divided by the Body Surface Area (BSA)*

*nPCcor - Non-linear Phase Contrast correction*

*Abs - Absolute*
